# Comprehensive Evaluation Method of Digital Economy Development Level Based on Complex Network Model

**DOI:** 10.1155/2022/4999178

**Published:** 2022-05-31

**Authors:** Xingjun Shi

**Affiliations:** Zhejiang Industry and Trade Vocational College, Wenzhou, Zhejiang 325002, China

## Abstract

In today's economic globalization and artificial intelligence modern society, the rapid economic growth mainly relies on the vigorous development of digital information technology. Simultaneously, digital technology promotes economic revolution and produces a digital economy, which has become an important concept of China's economic development. In order to more scientifically and accurately describe the development status of my country's industrial structure and digital economy industry, this paper takes each industry of the national economy as the research object and puts forward the idea of using complex network theory to study the relationship between input and output. Based on the complex network theory, this paper analyzes the interrelationships between various industries in the complex system. By referring to the relevant data of the national economic input and output in recent years, the input-output correlation network model between the various industries in the national economic system is established. Using the method of combining statistical analysis and comparative analysis, quantitative analysis and qualitative analysis, carry out detailed and in-depth statistical analysis from the input-output correlation network model and model attributes so as to point out the status and role of various industries in the national economy and so as to analyze and study the industrial structure of the national economy.

## 1. Introduction

With the development of the emerging economy, the digital economy has gradually entered people's field of vision and has received more and more attention. The scale of my country's digital economy continues to expand, and the innovative Internet, such as big data technology, information technology, and cloud computing, has further expanded the reach and service scope of the digital economy, becoming a key engine for promoting economic growth and driving high-quality economic development. The exchange of different elements between industries drives the interaction between various industries. The industrial sector of the national economy forms an industrial network model through the input-output relationship. The characteristics of the industrial network structure affect the evolution of the industrial structure. The emergence of the digital economy industry depends on the full development of the digital economy. The construction of its indicator system is based on traditional products and services, but it also covers the digital Internet economy and the advantages of the digital economy that it brings [1, 2].

On this basis, as so to better explore the development of the digital economy, this paper first combines the input-output correlation analysis with the complex social network model and then builds an input-output correlation model based on complex network theory so as to systematically explore the relationship and position of each industry from the overall level. The main network attributes of each industry, such as intensity distribution, agglomeration coefficient, etc., are analyzed, and the degree and impact of digital economy industries on other industries are discussed. Then, by referring to the compilation modus of the traditional financial inclusion index, combined with the current characteristics of my country's digital economy, using the analytic hierarchy process based on the coefficient of variation to determine the index weight, and establish an evaluation system [3]. On the basis of the overall index, the digital economy index is subdivided into three different dimensions: coverage breadth, depth of use, and degree of digital support services, which measures the development level of the digital economy from two macro- and microlevels and puts forward corresponding conclusions. The conclusions presented give reasonable suggestions.

## 2. Definition of Digital Economy Industry and Related Research Methods

This paper conducts a social network analysis on the characteristic structure and degree of correlation between the digital economy industry and other industries, which is not only a supplement and improvement of the theory in the digital economy field but also a new exploration of applying social network analysis methods to the digital economy industry. At the beginning of the whole, each industry is regarded as the research object, combined with the input-output modus in the national economy, the direct consumption coefficient is used as the weight to weigh the edges connected to each industry, and the strong correlation is extracted by setting the critical value, and then the strong correlation is constructed. An industrial complex network graph of relationships: using indicators such as network density, centrality, agglomeration coefficient, and block model, this paper analyzes the characteristics of my country's industrial development; by extracting digital economy-related industries, measuring the internal connection between digital economy industries and other industries, and exploring my country's overall and local perspectives. The characteristics of the industrial network also reveal the characteristics of the input-output correlation network of the industrial structure of my country's national economy [4–6]. Applying the complex network to the study of the industrial structure network is a new attempt in the economic system, and there are still many works. It can be further explored, and the above content also provides ideas for the development research of other industries. The research on enriching the digital economy industry and other industrial structures also has certain theoretical significance. This paper first studies the definition standards and development of the digital economy at home and abroad and then sets out from the actual situation in my country, breaks through the traditional perspective, from the perspective of the innovative digital economy, and scientifically and comprehensively establishes an indicator system that can summarize the digital economy from a multidimensional perspective. It is not only helpful to promote the deepening of digital economy research but also a useful supplement to the development of the traditional economy.

### 2.1. Research Status at Home and Abroad

Jaakkola et al. used the S-shaped curve to describe the spillover process of high-tech enterprises such as information and communication technology [7]. Some scholars explored the production efficiency of different industries in the United States and found that industrial productivity improvement is related to information and communication technology. It is relatively obvious that information and communication technology has a pulling effect on its related industries [8, 9], and there is a strong spillover effect between the information and communication technology industry and other industries, and it is the key engine for promoting economic growth [10], simultaneously, the information and communication technology manufacturing industry shows high heterogeneity, indicating that the information and communication manufacturing industry is more widely used in the industry. There are also scholars who hold different views on the role of the digital economy. Bart pointed out that although the digital economy has developed rapidly, it remains to be discussed whether it has played its greatest role [11]. Through further quantitative calculation, Chattopadhyay concluded that if the banking system lacks a digital economy, the national economy will lose 1% [12]. Demirguc and Klapper stated in the World Bank report that digital finance could help the poor to obtain deposits or borrowings at a fixed and reasonable ratio [13] and build a safer payment transaction environment by establishing a credit system. Indian economist Sarma draws on the construction modus of the United Nations Human Development Index (HDI) [14] by setting the availability and specific usage of financial services and banking penetration as the main indicators, using Euclidean distance and linear efficacy function to correlate. The combined modus reasonably measures the basic status of the digital economy in different countries [15–17].

According to the definition of the digital economy, the China Academy of Information and Communications Technology and Liu divide the digital economy into two parts: industrial digitization and digital industrialization [18], of which the industrial digitization part refers to the integration of other industries and digital technologies [6, 19]. An application can increase and improve the output and efficiency brought by the use of digital technology and digital products in other nondigital industrial sectors of the national economy; digital industrialization includes digital product production and digital technology innovation, mainly covering electronic information manufacturing, information communication industry, Internet industry, and software service industry. To explore the information and communication technology industry in-depth, Wen used the social network model to measure the degree of relatedness of the information technology industry [20–22] and innovation co-operation space and found that the local spatial organization innovation characteristics of the cluster innovation network society are obvious, and the innovation subjects in the cluster area are more inclined to cooperate with the innovation subjects in other cluster areas; the latter adopts the input-output model to obtain a new generation of information technology. The main relevant departments of the industrial chain are used to measure the degree of correlation between the industrial chains by using the average sweeping steps method [23, 24].

The research of domestic and foreign scholars on industrial correlation and the digital economy industry provides an important reference for this article. As a new economic form arising from the development of the information technology revolution, most scholars study the digital economy industry or application at the theoretical level. The regression method conducts an empirical analysis of the digital divide in different regions, while the research on the development of the digital economy industry and its degree of correlation with other industries is relatively rare, not in-depth enough, and lacks multilevel research. The research on the social network in foreign countries is earlier than that in China. A lot of explorations have been carried out in both theoretical and empirical aspects, and a series of rich research upshots have been obtained, providing important references and inspiration for the research of domestic industrial social networks.

### 2.2. Possible Innovations and Deficiencies of Existing Research

1. Innovation in research perspective. The digital economy industry is an emerging industry, so this paper takes the digital economy as a research perspective, which is an innovation. Drawing on the definition of the digital economy industry in other countries and considering the development status of my country's digital economy industry, the digital economy industry is defined as a digital technology-based industry. The sum of various economic activities is carried out to determine the main areas of my country's digital economy industry. This article compiles the index based on a large amount of microdata provided by Ant Financial Group and the relevant data from the statistical yearbook that can be found. The index not only covers the emerging digital economy industry but also integrates traditional economic businesses, such as banks. From the perspective of measuring the development status of the digital economy, it is reliable and representative. Not only does it provide a basis for government decision-making, but local governments can also use this index to judge the level of digital economic development in their region and learn from the development experience of other regions. Secondly, it provides a basis for decision-making reference for enterprises to make business decisions. Finally, the construction of a digital economy development index can also help to more accurately understand the development of the entire industry and increase the understanding of the digital economy.
2. Innovative research modus. Apply social network analysis to industrial structure analysis. The input-output method is widely used in the study of industrial structure, but it cannot visualize the closeness and concentration trend of interindustry relations and has certain limitations, and the social network analysis modus can make up for the shortcomings. Therefore, based on the input-output analysis method, this paper introduces the perspective of network analysis, which can dig deeper into the correlation of various industries and provide support for identifying the development status of the industrial structure. Through the construction of my country's industry association network, the role of my country's industry is analyzed, and it is divided into different sections to study its function and role. Simultaneously, taking the industrial system as a whole reveals the overall characteristics of my country's industrial development; simultaneously, the various industrial elements that make up the complex industrial network model also reveal the level and structure of the entire network and clearly explain the relationship between industries from the overall and local levels and the tightness of connections and the relationship between industry behavior across the network. The logarithmic efficacy function is used to carry out the dimensionless treatment of the index so as to maintain the stability of the index and alleviate the influence of extreme values. Finally, according to the characteristics of the digital economy index, the weighted arithmetic average modus is used to construct the digital economy index.
3. Due to the relative limitations of the data, the research on the current situation of industrial development in this paper only analyzes the year 2015, which cannot fully demonstrate the actual situation of the current industrial development. In addition, due to the limited academic level of individuals, there is also a lack of in-depth research on the mechanism and impact of industry associations and research on the prediction of industry development. In addition, due to the availability of data and the compatibility of data from different institutions, the research angle and indicators may be slightly single, and there is no in-depth research and exploration from a certain dimension.

## 3. Analysis of Network Characteristics of China's Digital Economy Industry

**Figure 1:**
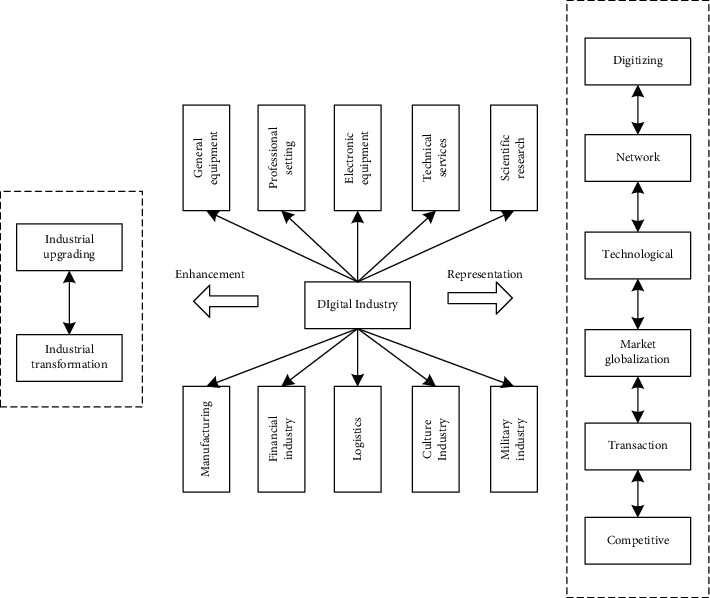
Digital industry structure chart.

The input-output method proposed by Leontief is the most commonly used modus in industrial linkage research. However, the input-output method can only reflect the relationship between industrial departments and cannot reflect the structural characteristics of industrial departments in detail, and the input-output analysis modus needs to meet basic assumptions such as homogeneity and proportionality. Therefore, based on the input-output table, this paper uses the social network analysis method to establish an industrial network model. It is possible to analyze further the relationship between the digital economy industry and other industries, as well as the overall structural characteristics of my country's industrial associations so as to provide support for the development of the digital economy industry. As shown in Figure 1, the digital economy plays an irreplaceable role in promoting the economic development of all walks of life, deepening economic reform, and promoting industrial upgrading by using mathematical thinking and digital technology and platforms.

### 3.1. Industrial Network Model Construction

The paper adopts the idea of input share to connect the edges of the strong correlation relationship with the direct consumption coefficient of the industry greater than the average value, weigths the edges with the direct consumption coefficient, and establishes the industrial network model *N* = (*V*, *E*, *W*), where *V* is the node industry, and *E* is the directed edge between nodes. It reflects the correlation between industries, and *W* = {*W*_*ij*_} is the weight, which represents the direct consumption coefficient of industry *j* to industry *i*.

(1) Determination of Nodes According to my country's inter-regional input-output table, my country is divided into 42 major departments. Therefore, the inter-regional industrial space network constructed in this paper has a total of 42 nodes.
(2) Determination of side and side rights, the input-output relationship between industries in each region is regarded as the edge of the network.The direct interregional industry correlation coefficient is introduced as the edge. The interregional industry direct correlation coefficient is introduced as the weight of the edge, and the critical value is set to determine the existence of the edge. Calculate the direct correlation coefficient equation (1) below. Among them, *X*_*j*_ is the output of *j* industry, *X*_*ij,*_, and it is the intermediate input of *i* industry to *j* industry. When constructing the industrial network model, the direction of input and output is considered. Therefore, for each node, there are actually four edges connected to it, node 1 is connected to four directed edges, and each of the edges has a different weight.
  (1) $$\begin{array}{c} a_{ij}=\frac{x_{ij}}{x_{j}}\left(i,j=1,2,\ldots ,n\right). \end{array}$$
(3) Theil index method

Theil index modus was first proposed by Theil in 1967. Its biggest advantage is that it can decompose the gaps between regions and within regions, which is more conducive to exploring regional development gaps and causes, and formulating more targeted policy. Therefore, it is more authoritative and convincing to select the Theil index to study the development gap of the national digital economy. The calculation method of the Theil index of the overall difference in the development of the national digital economy is shown in the following formula (2):

(2) $$\begin{array}{c} T=T_{b}+T_{w}=\sum_{k=1}^{k}{\text{del}}_{k}\log\left(\frac{{\text{del}}_{k}}{\left(n_{k}/n\right)}\right)+\sum_{k=1}^{k}{\text{del}}_{k}\left(\sum_{\text{i}\in g}^{k}\frac{{\text{del}}_{i}}{\text{del}}\log\frac{\left({\text{del}}_{i}/{\text{del}}_{k}\right)}{\left(1/n_{k}\right)}\right). \end{array}$$

*T*_*b*_ and *T*_*w*_ represent the digital economic development gap between regions and the digital economic development gap within the region, respectively, *n* represents the number of individuals, *K* represents *K* groups, and each group is *g*_*k*_ (*k* = 1, 2,…, *K*), the number of individuals in the *K* group *g*_*k*_ is *n*_*k*_, and del_*i*_ and del_*k*_ represent the digital economic development level of individual *i* and the overall digital economic development level of group *K,* respectively.

### 3.2. Analysis of the Overall Characteristics of the Industrial Network

The comprehensive index of the digital economy development level is the basic data for the study of regional differences in the development of the digital economy. The paper mainly starts from the three dimensions of infrastructure, digital transaction, and digital industry and establishes a comprehensive evaluation index system for the development level of the digital economy, as shown in Table 1. The comprehensive index modus measures the development level of my country's digital economy. The larger the obtained comprehensive development index of the digital economy, the higher the development level of digital economy, and vice versa, the lower the development level of the digital economy.

The paper uses the Theil index method to calculate the development level of the national digital economy. Overall differences, examining the differences in the development of digital economy between regions and within regions in my country's current situation, the upshots calculated by Theil index method are shown in Table 2. The upshots, respectively, draw a map of the regional differences in the digital economy and the internal differences of the seven regions. Figure 1 shows a map of the differences in the digital economy.

**Figure 2:**
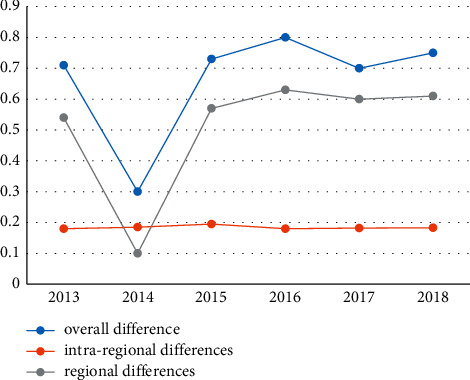
Map of regional differences in the digital economy.

It can be seen from Figure 2 that since 2013, the overall regional differences in the development level of my country's digital economy have shown a W-shaped trend. The overall difference showed an expanding trend, rising from 0.3014 in 2014 to 0.7770. Specifically, from 2013 to 2014, the overall regional difference in the development level of my country's digital economy showed a downward trend, from 0.7032 to 0.3014, and increased significantly from 2014 to 2016, from 0.7032 to 0.3014. 0.3014 rises to 0.8354, which decreased from 2016 to 2017, from 0.8354 to 0.7215, and then began to rise again, reaching 0.7770 in 2018, showing an overall upward trend. The difference in digital economy development between regions is basically similar to the overall regional difference in the level of digital economy development and has a strong positive correlation with the overall regional difference in the level of digital economy development. Differences and differences in digital economic development between regions have the opposite trend, showing a downward trend as a whole, from 0.1779 in 2013 to 0.1538 in 2018, but the change is small. Specifically, from 2013 to 2015, the difference in the development of the digital economy within the region showed an upward trend, rising from 0.1779 in 2013 to 0.1918 in 2015, and began to decline slightly from 2016, and then began to decline overall.

Judging from the differences in the development of the digital economy within the seven regions of my country, at the beginning of the whole, the difference in the development of the digital economy within the North China region is the largest. Although there has been a certain decline in recent years, it is still the highest among all regions. Second, the development of the digital economy in all regions of my country has shown a downward trend in recent years. Third, the differences in the development of digital economy in Northeast and North China are basically the same, and the development of digital economy in Northwest and Southwest regions shows a consistent trend. The impact of intra-regional differences and inter-regional differences was on overall differences and the impact on the development of the digital economy. This paper calculates the contribution rate of regional differences and intraregional differences to the overall differences in the development of the digital economy, as shown in Table 3.

## 4. Comprehensive Evaluation of Digital Economy Development in Various Regions of China

To further explore the reasons for the regional differences in the development of my country's digital economy, to put forward more effective policy suggestions for the current situation of the regional differences in the development of my country's digital economy, the paper uses the grey correlation modus to discuss the main reasons that affect the development of my country's digital economy, to narrow the country's digital economy and it shows the regional differences in the development of the digital economy and more effective suggestions for realizing the coordinated development of the national digital economy. According to the actual situation of my country's digital economy development, the paper believes that the reasons for the regional differences in the development of the digital economy can be mainly classified into three aspects: digital foundation, digital transaction, and digital industry. According to the calculation upshots of the grey correlation model, the following conclusions are drawn: Among them, the digital foundation has the highest degree of relevance, and its relevance ranks first, followed by digital transactions and digital industries, showing a ranking structure of digital foundation >digital transactions >digital industries.

Specifically, at the beginning of the whole, among all the influencing factors, the digital foundation has the greatest impact on the differences in the development of the national digital economy and is the main reason for regional differences. In recent years, with the development of the digital economy, various regions have accelerated the construction of digital economic infrastructure. However, due to the different levels of economic development in various regions, there will be a large gap in the investment in digital economy infrastructure, resulting in a large gap in the construction level of digital economy infrastructure in various regions, and infrastructure is the basis for the development of the digital economy. As a result, the development gap of the digital economy in various regions has shown an increasingly obvious trend. The second is the impact of digital transactions and digital industries on the development gap of the regional digital economy. Both digital transactions and digital industries are based on the Internet, breaking through geographical restrictions to a certain extent. Still, simultaneously, the economic development level of each region will also affect digital transactions and digital industries. Therefore, the two are important to the regional differences in the development of the national digital economy.

## 5. Conclusion and Recommendation

According to the previous research, the new technological revolution centered on the new generation of information technology has promoted the digital economy; it will give birth to many new industries. The development of the digital economy will expand the scope of production possibilities and accelerate industrial transformation and upgrade and promote high-quality economic development. Digital technology spawns new innovation models and infuses technological innovation with new impetus. This shows that taking advantage of the strategic opportunities brought by digital transformation accelerates the update of innovative ideas and it is particularly important to foster digital transformation, develop new R&D institutions, and optimize the policy environment for innovation.

### 5.1. Summary

1. The communication equipment, transportation equipment, computer, and other electronic equipment industries are in the central position. This may be because the tertiary industry, such as the transportation industry itself, has the function of linking other industries. This industry advantage is inherently unique. Therefore, as so to develop the regional economy, the government should pay attention to the close connection between industries. However, the internal structure of the tertiary industry has obvious hierarchical differentiation. Among them, the wholesale, retail industry, financial industry, transportation, warehousing, and postal industry have high centrality and occupy a dominant position in the entire industrial system. In contrast, the communication equipment, computer, and other electronic equipment manufacturing industries are ranked lower in emerging service industries such as high-tech industries, and almost all the resources of these industrial subgroups are used for internal consumption, only for internal industrial development. Provide resource support, resulting in a relatively unbalanced industrial structure.
2. The digital economy has realized the possibility of rapid economic development for economically backward regions and also laid the foundation for some middle-aged job seekers to obtain digital economic services with wider coverage and greater use depth. It is imperative to become high-tech intellectual talents. Physical talents in traditional industries will gradually be eliminated by society. In addition, the practice of the digital economy in recent years also shows that digital mobile payment technology provides a relatively strong ability to reach customers. With the advent of the era of intelligence, the integration of the digital economy into people's lives will only become deeper and more common, sharing high-quality resources for education and medical assistance in different regions. For remote areas, increasing the coverage of the digital economy and reducing the cost of use are the top priorities for everyone; and if the digital economy is limited to developed areas, it will not only lead to a widening gap between the rich and the poor but also lead to the occurrence of unbalanced social structure in the long run.

### 5.2. Prospect

1. According to the previous research, industries with high digital economic strength are better at obtaining innovative resources from other industries to further expand the strength of the industry but neglect to build their own influence radiation range. As an agglomeration industry of digital economic resources, it should exert its own influence from two aspects. At the beginning of the whole, within the subgroup, give full play to the influence of the core industry of the digital economy and export resources to the original subgroup members to improve the subgroup. The overall strength of the group's internal network enhances the stability of the industrial network structure and builds a foundation for the digital economic relationship network. Simultaneously, it is necessary to give full play to the role of the intermediary of the sub-groups, enhance the output of the superior innovation resources of the subgroups to the sub-groups, actively explore more available innovation revenue resources in the industry, and strive to expand the sub-groups while improving their own innovation strength. The scale of the group, such as the transportation industry, can be further combined with the digital economy to develop services such as robot home delivery or intelligent rail transit so that the core industry can play its own advantages while driving the integrated development of other service industries.
2. The industrial age before the digital age emphasized economic scale; that is, the production cost of industry decreased with the increase of a single production scale, and the cost reduction could only depend on the increase of production quantity, which is a kind of decentralized development. Economic model: at present, the digital economy pays more attention to the development of a coordinated economy. In other words, under the condition of open resources, by strengthening the overall planning between different organizations, group development can bring greater economic benefits than self-development. The different characteristics of different regions in my country, such as industrial structure, innovation resource distribution, and digital resource endowment, also determine the unique locational environmental advantages, digital resources, scientific, technological innovation resources, and other advantages of cities such as Beijing and Shanghai, which make these cities have advantage point for an internationally competitive digital economy.

## Figures and Tables

**Table 1:** Comprehensive index system of digital economy development level.

Primary indicator	Secondary indicator	Measurement method	Unit
Base installation	Cable length	Cable length per square kilometer	kilometer
	Computer users	Every 100 households use a computer	Platform
	Internet access volume	Internet broadband access users per 10,000 people	Household

Digital transaction	Internet usage	per 10,000 mobile internet users	Household
	The integration of digital economic entities	The number of enterprises with e-commerce transactions per 10,000 friends	Individual
	E-commerce sales	E-commerce sales per 10,000 people	Billion

Digital industry	E-commerce purchases	E-commerce purchases per 10,000 people	Billion
	Digital industry investment	Fixed asset investment per 10,000 people	Billion
	Digital industry human resources	Proportion of employees in information transmission services	%
	Digital industry turnover	Information service income per 10,000 people	Million

**Table 2:** Calculation results of the Theil index for the development level of the digital economy in the interval.

Years	All difference index	Intraregional difference index								Regional difference index
		North	Northeast	East	Central	South	Southwest	Northwest	Intraregional	
2013	0.703	0.449	0.050	0.273	0.075	0.165	0.036	0.031	0.177	0.525
2014	0.301	0.433	0.196	0.231	0.195	0.117	0.028	0.043	0.188	0.113
2015	0.757	0.506	0.075	0.247	0.048	0.183	0.076	0.062	0.191	0.565
2016	0.835	0.496	0.029	0.134	0.033	0.161	0.044	0.061	0.152	0.682
2017	0.721	0.461	0.012	0.176	0.029	0.166	0.065	0.041	0.153	0.567
2018	0.777	0.467	0.026	0.185	0.025	0.148	0.069	0.037	0.158	0.623

**Table 3:** The contribution rate of regional differences and intraregional differences to the overall differences in the digital economy development.

Years	Intraregional contribution rate	Contribution rate between regions
2013	0.253001901	0.746998099
2014	0.624425624	0.375574376
2015	0.253257645	0.746742355
2016	0.182713242	0.817286758
2017	0.212899485	0.787100515
2018	0.197951304	0.802048696

## Data Availability

The dataset can be accessed upon request.
